# Structural characterization and bioactivity evaluation of water-extractable polysaccharides from chickpeas (*Cicer arietinum* L.) seeds

**DOI:** 10.3389/fnut.2022.946736

**Published:** 2022-07-28

**Authors:** Yingying Zhu, Baoqing Dun, Zhenxing Shi, Yuanji Wang, Li Wu, Yang Yao

**Affiliations:** ^1^Institute of Crop Science, Chinese Academy of Agricultural Sciences, Beijing, China; ^2^Henan Key Laboratory of Cold Chain Food Quality and Safety Control, College of Food and Bioengineering, Zhengzhou University of Light Industry, Zhengzhou, China; ^3^School of Food Science and Technology, Henan University of Technology, Zhengzhou, China

**Keywords:** chickpea, polysaccharides, chemical structural, antioxidant, immunoregulatory

## Abstract

Two water-extractable polysaccharide fractions designated as CWP (7. 37 × 10^5^ Da) and CWP-0.2 (1.58 × 10^4^ Da) were isolated and purified from chickpea (*Cicer arietinum* L.) seeds. The chemical structure of the two polysaccharides was characterized by various methods. Monosaccharide composition and methylation analysis showed that CWP was composed of Man and Glc in a molar ratio of 44.6:55.4, and CWP-0.2 was composed of Rha, Ara, Man, Glc, and Gal in a molar ratio of 10.6:23.3:5.2:4.9:56. Further structural characterization indicated that the main chain connection of CWP was → (2-β-d-Fru*f*-1) n →, and the main chain connection of CWP-0.2 was explored as → 2,4)-α-l-Rha*p*-(1 → 3)-α-d-Gal*p*-(1 → with the branched chain of → 2,4)-α-l-Rha*p*-(1 → o-4. Besides, both CWP and CWP-0.2 had antioxidant and immunoregulatory activity *in vitro*, through scavenging DPPH· and ABTS·^+^ as well as stimulating production of NO, IL-6, TNF-α and MCP-1 in RAW 264.7 macrophages. CWP-0.2 revealed significantly higher bioactivity than CWP.

## Introduction

Chickpea (*Cicer arietinum* L.) is the third most important legume in the world (1), with a long planting and application history in China, and is especially used in traditional Chinese Uygur medicine (2). Chickpea possesses high-quality starch, protein, fat and dietary fiber (3), and plays an important role in human diets. Previous studies have shown that chickpea possesses various biological activities, such as antioxidant (4), anti-inflammatory (5), amylase inhibitor (6), and angiotensin I-converting enzyme (ACE) inhibitory (7) activities.

Plant polysaccharides are natural high-macromolecular polymers with diverse potential medicinal characteristics and biological functions, with low cytotoxic side effects (8–12). Thus, they have received increased interests in recent years. Previous studies reported that chickpeas hulls are good source of plant polysaccharides with excellent functional properties and biological activities (4, 7, 13). To explore structural characteristics of polysaccharides from chickpea hulls (CHP), Ye et al. (4) optimized the extraction conditions for isolation of CHP, and reported the molecular weight and monosaccharide composition (4). Mokni Ghribi et al. (7) and Akhtar et al. (13) further explored the structure of CHP with CP/MAS ^13^C nuclear magnetic resonance spectroscopy (NMR), and demonstrated the presence of 1,4-d-galacturonan with methyl-esterified carboxyl group, 1,4-α-d-galactopyranosyluronan and methyl carbons of the methyl ester (COOCH_3_) (7, 13). As reported, polysaccharides are not only existed in chickpeas hulls, the cell walls of endosperm also contain various plant polysaccharides. However, the recent studies mainly focused on the CHP, researches on the structure of polysaccharides from chickpea seeds are still limited. Besides, more detail information on the structural characteristics of plant polysaccharides can be explored from the ^1^H-NMR, Dept135, HSQC, HHCOSY, and HMBC 2 D spectral data (14). A comprehensive understanding of the structures of chickpea polysaccharides is remained to be studied. In addition, the relationship between the bioactivity and chemical structure of chickpea polysaccharide is ambiguous and needs to be explored.

Therefore, the purpose of the present study was to obtain purified water-extracted polysaccharides from chickpea seeds with ion-exchange chromatography and to further characterize their chemical structure; to evaluate their antioxidant activity and immunoregulatory activity *in vitro*; and to analyze the correlation between the chemical structure and bioactivity of water-extracted chickpea polysaccharides.

## Materials and methods

### Materials and chemicals

Chickpea seeds were obtained from the National Gene Bank (Beijing, China). DEAE Sepharose Fast Flow was obtained from GE Healthcare Bio-Sciences Co. (Piscataway, NJ, USA). Dextran with different molecular weight (5,000–670,000 Da), griess reagent, arabinose (Ara), rhamnose (Rha), mannose (Man), galactose (Gal), glucose (Glc), 1,1-diphenyl-2-picrylhydrazyl (DPPH), 2,2'-azinobis-(3-ethyl-benzothiazolin-6-sulfonic acid) diammonium salt (ABTS), and 3-(4,5-dimethylthiazol-2-yl)-2,5-diphenyltetrazolium bromide (MTT) were purchased from Sigma-Aldrich (St. Louis, MS, USA). RPMI 1640 media, lipopolysaccharide (LPS), phosphate-buffered saline (PBS), and fetal bovine serum (FBS) were obtained from Gibco BRL Life Technologies (Thermo Fisher Scientific, NY, USA). Raw murine macrophage 264.7 (RAW 264.7) cells were purchased from the Cell Resources Center of the Chinese Academy of Sciences (Shanghai, China). OptEIA ELISA kits for tumor necrosis factor-α (TNF-α), MCP-1 and interleukin-6 (IL-6) were purchased from BD Biosciences (San Diego, CA, USA). A PathScan Antibody Array Kit was purchased from Cell Signaling Technology (Shanghai, China). All other chemicals and solvents used were analytical grade, unless otherwise specified.

### Isolation and purification

The chickpea water-extracted crude polysaccharides (CWCP) from chickpea seeds were obtained according to the method of Yao et al. (15). Briefly, chickpea seeds were ground and passed through a 0.5-mm sieve, and the chickpea flour was pre-extracted with 95% ethanol (1:10 w/v) for 3 days to remove fat and small molecules. The residue was extracted twice with distilled water (1:20 w/v) at 90°C for 4 h. After centrifugation (4,000 g, 15 min), the supernatant was collected and deproteinated by the Savag method (16). The CWCP was obtained by freeze-drying. Then, CWCP (210 mg) was dissolved in distilled water (7 mL) and centrifuged at 10,000 g for 10 min, and then the supernatant was loaded onto the ÄKTA explorer 100 purification system with a DEAE Sepharose Fast Flow column. The column (100 cm × 2.6 cm) was first eluted with ultrapure water and then stepwise eluted with 0 to 2.0 M NaCl at a flow rate of 5 mL/min. The fractions (10 mL/tube) were collected using an automatic fraction collector, and 1 mL was removed from each tube and mixed with 1 mL of distilled water, 0.5 mL of 6% phenol solution and 5 mL of sulfuric acid. The absorbance was determined at 490 nm after reaction for 20 min using an ELISA reader (MULTISKAN GO, Thermo Fisher Scientific, USA). Four final fractions were collected, dialyzed and lyophilized, namely, CWP, CWP-0.2, CWP-0.5 and CWP-2, with yields of 35.10, 27.32, 0.83, and 0.98%, respectively. Due to the low yields of CWP-0.5 and CWP-2, only CWP and CWP-0.2 were further used to analyze the purity, chemical structure and biological activity. The purities of CWCP, CWP and CWP-0.2 were determined by the phenol-sulfuric acid method (17).

### Analysis of molecular weight

The high-performance gel permeation chromatography (HPGPC) method was used to analyze the molecular weight (18). The sample and dextran with different molecular weight were prepared at 5 mg/mL and centrifuged (12,000 rpm, 10 min). The supernatant was filtered through a micropore filter with an injection volume of 20 μL. The high-performance liquid chromatography (HPLC) analysis system (Shimadzu LC-10A) was equipped with a BRT102 gel column (8 × 300 mm) (Borui Saccharide, Biotech. Co. Ltd.). The experimental conditions were as follows: column oven temperature: 40°C, flow rate: 0.8 mL/min, and mobile phase: ultrapure water.

### Analysis of monosaccharide composition

Gas chromatography (GC) was used for the identification and quantification of monosaccharide components. CWP and CWP-0.2 (5 mg/mL) were hydrolysed with trifluoroacetic acid (2 M) at 120°C for 4 h. The released monosaccharides were converted into trimethysilylated derivatives and analyzed by GC on an Agilent 6890 instrument (Agilent Technologies, Santa Clara, CA, USA) equipped with an HP-5MS column (0.25 mm × 30 m × 0.25 μm) and were determined using a flame ionization detector (FID). The column temperature and other parameters were set according to a previous method (19).

### Methylation analysis

Methylation analysis was performed by a previously described method (20). Briefly, 10 mg of CWP or CWP-0.2 and 2 mg of NaOH were dissolved in 100 μL of DMSO, and then methyl iodide was added to the reaction. Methylated polysaccharide was taken, and 2 M trifluoroacetic acid (1 mL) was hydrolysed for 90 min and then evaporated to dryness by a rotary evaporator. The residues were hydrolysed with 10 mL of 2 M trifluoroacetic acid (10 mL) at 100°C for 8 h, the hydrolysates were dissolved in 4 mL of cold 1% (w/w) NaOH, and then 3 mL of toluene was added. Samples were concentrated under reduced pressure and evaporated to dryness. The acetylated product was dissolved in 3 mL of chloroform and transferred to a separatory funnel. The chloroform layer was dried with anhydrous sodium sulfate, and the volume was fixed at 10 mL. The analysis was performed using a Shimadzu GCMS-QP 2010 gas chromatography-mass spectrometer.

### Nuclear magnetic resonance spectroscopy (NMR) spectroscopic analysis

In a D_2_O solution at 20°C, Bruker Avance 600 and Bruker Avance 500 NMR spectrometers (Bruker, Ettlingen Germany) were used to record proton NMR and ^13^C APT NMR spectra (operating frequencies of 1H: 600.1 MHz and 499.8 MHz, operating frequencies of ^13^C: 150.9 MHz and 125.7 MHz). MestReNova 10.0 (Mestrelab Research, Santiago de Compostela, Spain) and Origin 6.0 (Microsoft Windows, Redmond, USA) were used to analyse the data and generate NMR spectra. ^1^H and ^13^C spectra, Dept135, HSQC, HHCOSY and HMBC spectra and CWP and CWP-0.2 spectra were recorded at 30 MHz with an MBC spectrometer (Bruker, Rheinstetten, Germany). Tetramethoxysilane was used as an internal standard.

### Assay of antioxidant activity

#### DPPH·radical scavenging activity

The DPPH·radical scavenging capability of CWP and CWP-0.2 was evaluated according to our previous method with slight modification (21). CWP and CWP-0.2 were dissolved in distilled water at different proportions (0.5–2.5 mg/mL), and 2 mL of the polysaccharide solution was added to tubes mixed with 2 mL of DPPH· solution (0.2 mM). The mixture was co-incubated for 30 min in the dark at room temperature. The absorbance of the resulting solution was detected at 517 nm, and Trolox was used as a positive control.

Scavenging rate (%) = [1–(A_i_-A_j_)/A_0_] × 100 (I)

where A_0_ is the absorbance of the control (distilled water instead of samples), A_i_ is the absorbance in the presence of the sample and DPPH·, and A_j_ is the absorbance of the sample blank (ethanol instead of DPPH·).

#### ABTS·^+^ radical scavenging activity

The ABTS·^+^ radical scavenging capability of CWP and CWP-0.2 was measured using the reported method with some modifications (22). The ABTS·^+^ reaction solution was prepared using a balanced mixture of ABTS (7.4 mM) and potassium persulfate (2.6 mM), and the mixture was incubated at 25°C for 12 h. The absorbance was adjusted to 0.7 ± 0.02 at 734 nm. For each sample, 0.4 mL of CWP and CWP-0.2 (0.5–2.5 mg/mL) were mixed with 1.6 mL of ABTS^+^, and the mixture was co-incubated for 6 min in the dark at room temperature. The absorbance was measured at 734 nm, and Trolox was used as a positive control.

Scavenging rate (%) = [1–(A_i_-A_j_)/A_0_] × 100 (II)

where A_0_ is the absorbance of the control (distilled water instead of samples), A_i_ is the absorbance in the presence of the sample and the ABTS^+^ reaction solution, and A_j_ is the absorbance of the sample blank (PBS instead of the ABTS^+^ reaction solution).

The free radical scavenging activity was expressed as trolox antioxidant equivalent capacity (TAEC, μM/g).

### Assay of immunological activity

#### Cell cultures and treatment

The cell culture followed a published procedure with slight modification (23). Mouse RAW 264.7 macrophage cells were cultured in RPMI 1640 medium supplemented with 10% FBS, 1% streptomycin and 1% penicillin. Cells were seeded in 96-well tissue culture plates at a density of 2.5 × 10^6^ cells per well, with various concentrations of CWP and CWP-0.2 (20, 40, 60 μg/mL) or LPS (1 μg/mL) as a positive control group treated with RPMI 1640 medium instead of sample. The 96-well tissue culture plates were incubated at 37°C in 5% CO_2_/95% air for 24 h, and NO, MCP-1, TNF-α, and IL-6 levels in the culture medium were measured to determine the production of cytokines.

#### Quantification of NO, TNF-α, MCP-1, and IL-6

The production of NO by 2.5 × 10^6^ cells/well in a 96-well tissue culture plate induced by 20, 40, and 60 μg/mL CWP and CWP-0.2 was determined after 24 h. TNF-α, MCP-1, and IL-6 concentrations in the supernatants from cell cultures were measured using ELISA kits according to the manufacturer's instructions. NO production was analyzed as the accumulation of nitrite in the 96-well tissue culture plate, determined with Griess reagent. Briefly, culture supernatant (50 μL) was pipetted from the 96-well tissue culture plate and mixed with 50 μL of Griess reagent. After incubation for 15 min in a cell incubator, the absorbance was measured using a SpectraMax 384 plus ELISA reader (Molecular Devices, Sunnyvale, CA, USA) at a 540 nm wavelength using Maxpro 6.2.1 software (Molecular Devices) (24). The concentration of nitrite was calculated based on a standard curve of sodium nitrite (0–100 μM).

#### Statistical analyses of data

All data are expressed as the mean ± standard deviation (SD). Statistical analysis was performed using SPSS (version 17.0). ANOVA with Dunnett's *post hoc* test was used to determine the significant differences between group means at *p* < 0.05. All analyses were processed at least three times.

## Results and discussion

### Purity and molecular weight

**Figure 1:**
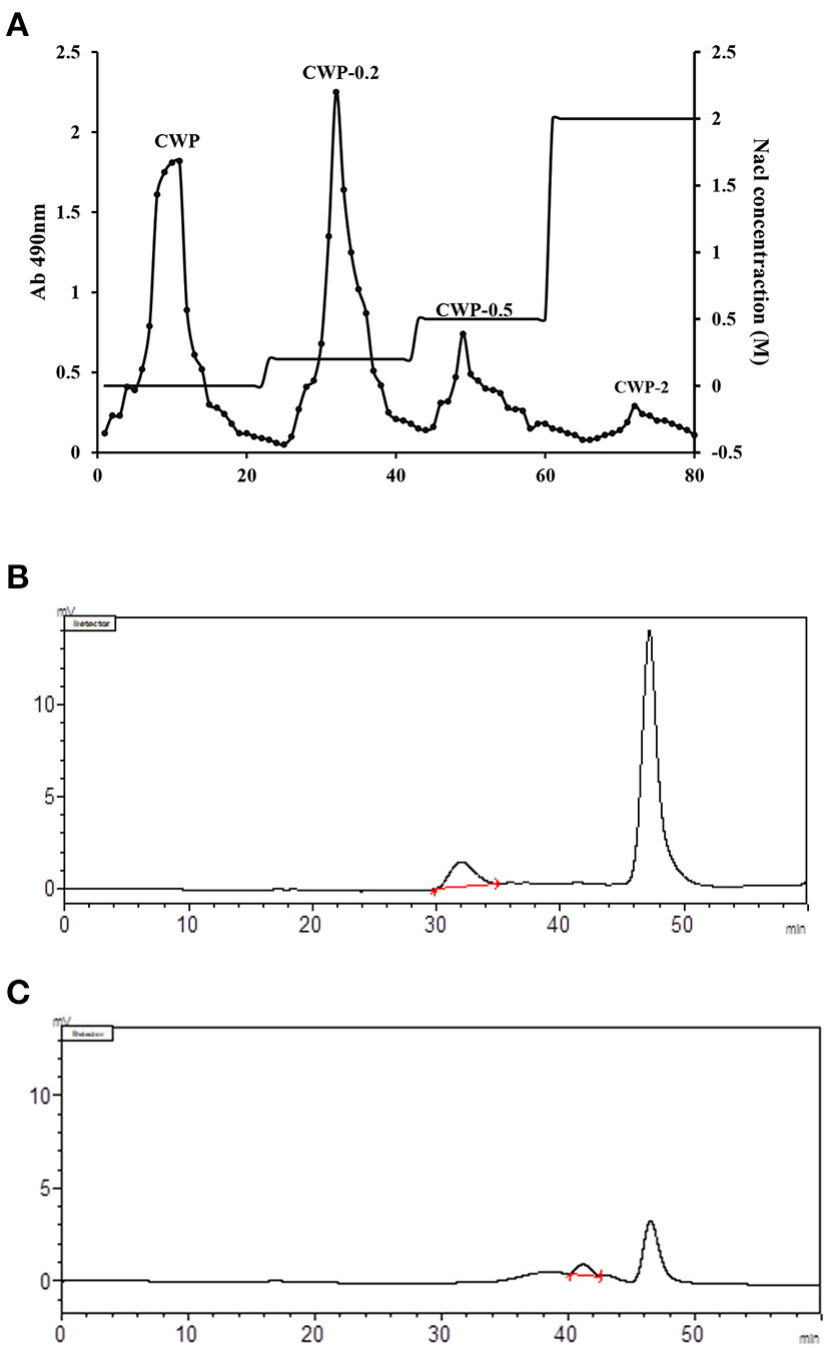
**(A)** Elution curve of chickpea polysaccharide on a DEAE Sepharose Fast Flow column. The crude polysaccharide was dissolved in distilled water and applied to the column, with elution with distilled water and NaCl (0.0–2.0 M). The eluent solution was collected and the carbohydrate content of the collected fraction was monitored using the phenol-sulfuric acid method. GPC chromatogram of **(B)** CWP and **(C)** CWP-0.2 for molecular weight determination with the size exclusion method Chromatography (SEC).

The purity of CWCP isolated from chickpea seeds was 80.34%. Chromatographic purification enabled two required fractions to be collected, and the purities of CWP and CWP-0.2 were 92.26 and 94.45%, respectively (Table 1). As shown in Figure 1A, CWP and CWP-0.2 showed single and symmetrical peaks, and the higher peaks, at 47 min, were NaCl in the mobile phase. The analysis indicated that the average molecular weights (Mw) of CWP and CWP-0.2 were 7.37 × 10^5^ Da and 1.58 × 10^4^ Da, respectively (Figures 1B,C; Table 1). Previous studies have uncovered that the molecular weight of polysaccharides extracted from chickpea hulls is 7.8 × 10^5^ Da to 3.1 × 10^6^ (4, 13). The present results showed that molecular weights of polysaccharides extracted from chickpea seeds were lower than that from chickpea hulls.

**Table 1:** Purity, molecular weight, and monosaccharids composition of CWP and CWP-0.2.

**Polysaccharides**	**Purity (%)**	**Molecular weight (Da)**	**Monosaccharids composition ratios**	
			**Monosaccharids**	**Ratio (mol%)**
CWP	92.26 ± 1.38	7.37 × 10^5^	Man	44.6
			Glc	55.4
CWP-0.2	94.45 ± 2.01	1.58 × 10^4^	Rha	10.6
			Ara	23.3
			Man	5.2
			Glc	4.9
			Gal	56

### Monosaccharide compositions and linkage analysis

CWP consisted of Man and Glc with a molar percent of 44.6:55.4. CWP-0.2 was composed of Rha, Ara, Man, Glc, and Gal with a molar percent of 10.6:23.3:5.2:4.9:56, respectively (Table 1). The molar percentages of Gal are large (more than 50%) in CWP-0.2. Ye et al. (4) uncovered the monosaccharide compositions of polysaccharides from chickpea, which consisted of Man, Rha, GalA, Glc, Gal and Ara, with a molar ratio of 0.03:0.43:0.06:0.06:0.43:0.11; CHPS-2 was composed of Man, Rha, GalA, Gal, Xyl, and Ara, with a molar ratio of 0.03:0.22:0.17:0.43:0.06:0.09; and CHPS-3 consisted of Rha, GalA and Gal, with a molar ratio of 0.34:0.08:0.57 (4). Akhtar et al. (13) also analyzed the monosaccharide compositions of CHPS, in which the molar percentages of galacturonic acid and galactose were 42.17 and 23.15%, respectively (13). Thus, our results were mutually confirmed.

To determine the linkage types, CWP and CWP-0.2 were subjected to methylation analysis (Table 2). The results showed that the main glycosidic bonds of CWP and CWP-0.2 were different. For CWP, the main glycosidic bonds were → 1)-Glc*f*-(2 →, → 1)-Man*f*-(2 → and Glc*p*-(1 →. The results of monosaccharide composition showed that CWP was composed of mannose and glucose. Whereas, the methylation results suggested that CWP was composed of fructan, because fructose was a ketose, it would be isomerized into mannose and glucose in the reduction process. For CWP-0.2, 11 glycosidic bonds were detected, and the main glycosidic bonds were Gal*p*-(1 →, → 3)-Gal*p*-(1 → and → 2, 4)-Rha*p*-(1 →. The se results were almost consistent with the ratio of monosaccharide. For the → 5)-Ara*f*-(1 → and → 4)-Ara*p*-(1 → in CWP-0.2, → 5)-Ara*f*-(1 → was common in polysaccharide, while → 4)-Ara*p*-(1 → usually appeared in the form of xyl*p*1–4. Therefore, we compared the results of monosaccharide composition and it was arabinose not xylose. There was no doubt that two forms of arabinoside bonds existed, isomerism. The same phenomenon was reported in a previous study (25).

**Table 2:** Monosaccharide linkage analysis of CWP and CWP-0.2 (molar ratios %).

**Polysaccharides**	**RT**	**Methylated sugar**	**Mass fragments (m/z)**	**Ratios**	**Type of linkage**
CWP	19.761	2,3,4,6-Me4-Glc*p*	43,71,87,101,117,129,145,161,205	16.04	Glc*p*-(1 →
	24.583	3,4,6-Me3-Man*f*	43,71,87,99,101,129,145,161,189	40.51	→ 1)-Man*f*-(2 →
	24.754	3,4,6-Me3-Glc*f*	43,71,87,99,101,129,145,161,189	43.43	→ 1)-Glc*f*-(2 →
CWP-0.2	9.569	2,3,4-Me_3_-Ara*f*	43,71,87,101,117,129,145,161	8.2	Ara*f*-(1 →
	14.691	2,3-Me_2_-Ara*f*	43,71,87,99,101,117,129,161,189	8.2	→ 5)-Ara*f*-(1 →
	14.967	2,3-Me_2_-Ara*p*	43,71,87,99,101,117,129,161,189	6.9	→ 4)-Ara*p*-(1 →
	17.591	2,3,4,6-Me_4_-Gal*p*	43,71,87,101,117,129,145,161,205	12.4	Gal*p*-(1 →
	18.901	3-Me_1_-Rha*p*	43,87,101,117,129,143,159,189	10.6	→ 2,4)-Rha*p*-(1 →
	20.846	2,4,6-Me_3_-Man*p*	43,71,87,99,101,129,145,161,189	8.2	→ 2)-Man*p*-(1 →
	21.143	2,3,6-Me_3_-Gal*p*	43,87,99,101,113,117,129,131,161,173,233	7.9	→ 4)-Gal*p*-(1 →
	21.436	2,3,6-Me_3_-Glc*p*	43,87,99,101,113,117,129,131,161,173,233	7.3	→ 4)-Glc*p*-(1 →
	22.241	2,4,6-Me_3_-Gal*p*	43,87,99,101,117,129,161,173,233	10.7	→ 3)-Gal*p*-(1 →
	24.49	2,3,4-Me_3_-Gal*p*	43,87,99,101,117,129,161,189,233	9.6	→ 6)-Gal*p*-(1 →
	29.619	2,4-Me_2_-Gal*p*	43,87,117,129,159,189,233	10	→ 3,6)-Gal*p*-(1 →

Data are expressed as mol % and represent the mean of three analysis. The PMAA derivative of a 5-linked-L-arabinofuranosyl^*^ residue 1,4,5-Tri-O-acetyl-1-deuterio-2,3-di-O-methyl-D-arabinitol; The PMAA derivative of a 4-linked-L-arabinopyranosyl residue 1,4,5-Tri-O-acetyl-1-deuterio-2,3-di-O-methyl-D-arabinitol.

### NMR spectroscopy

**Figure 2:**
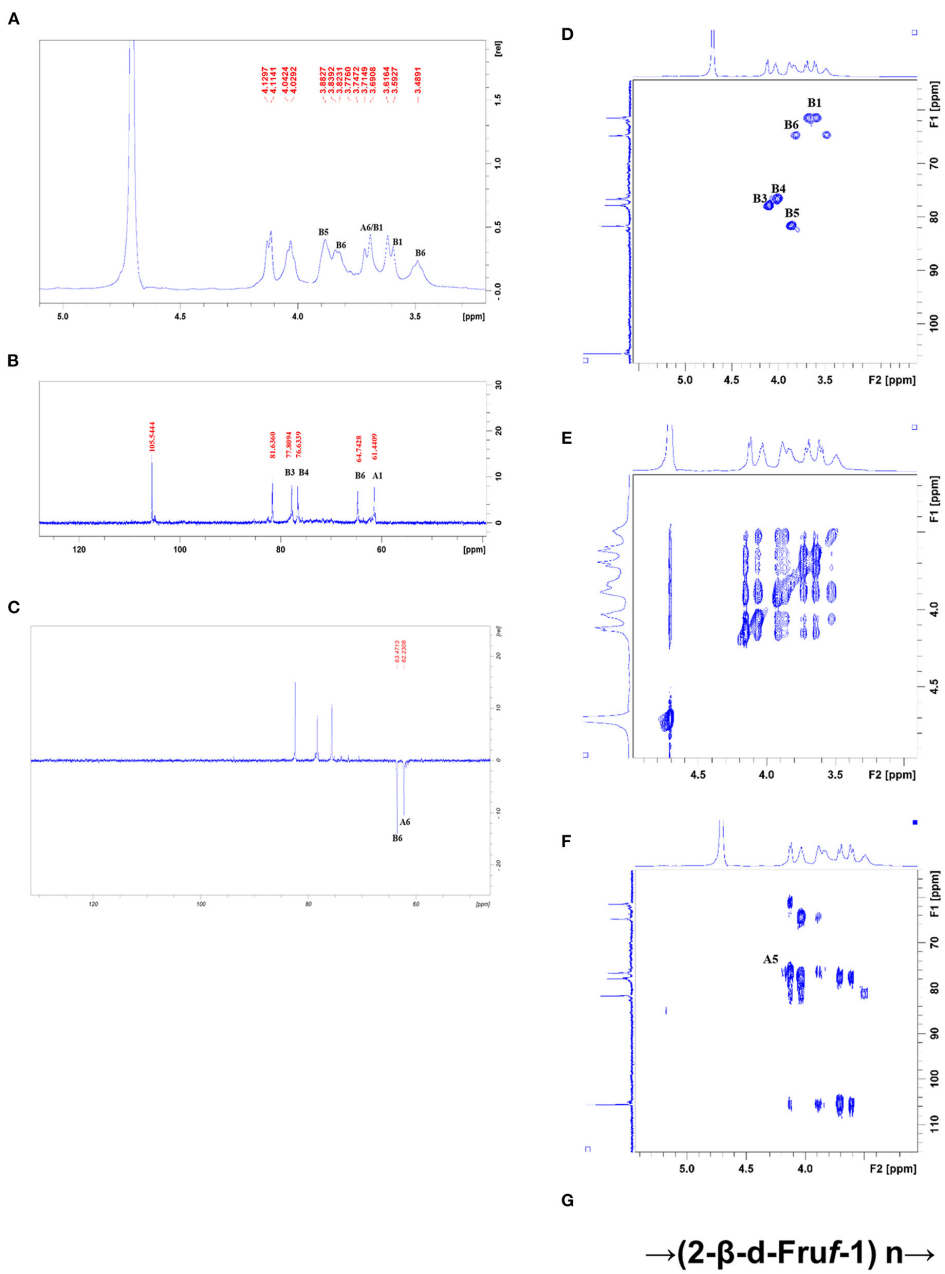
**(A)** ^1^HNMR, **(B)** ^13^C NMR, **(C)** DEPT-135, **(D)** HSQC, **(E)** HHCOSY, **(F)** HMBC spectra, and the structural formula **(G)** of CWP.

**Figure 3:**
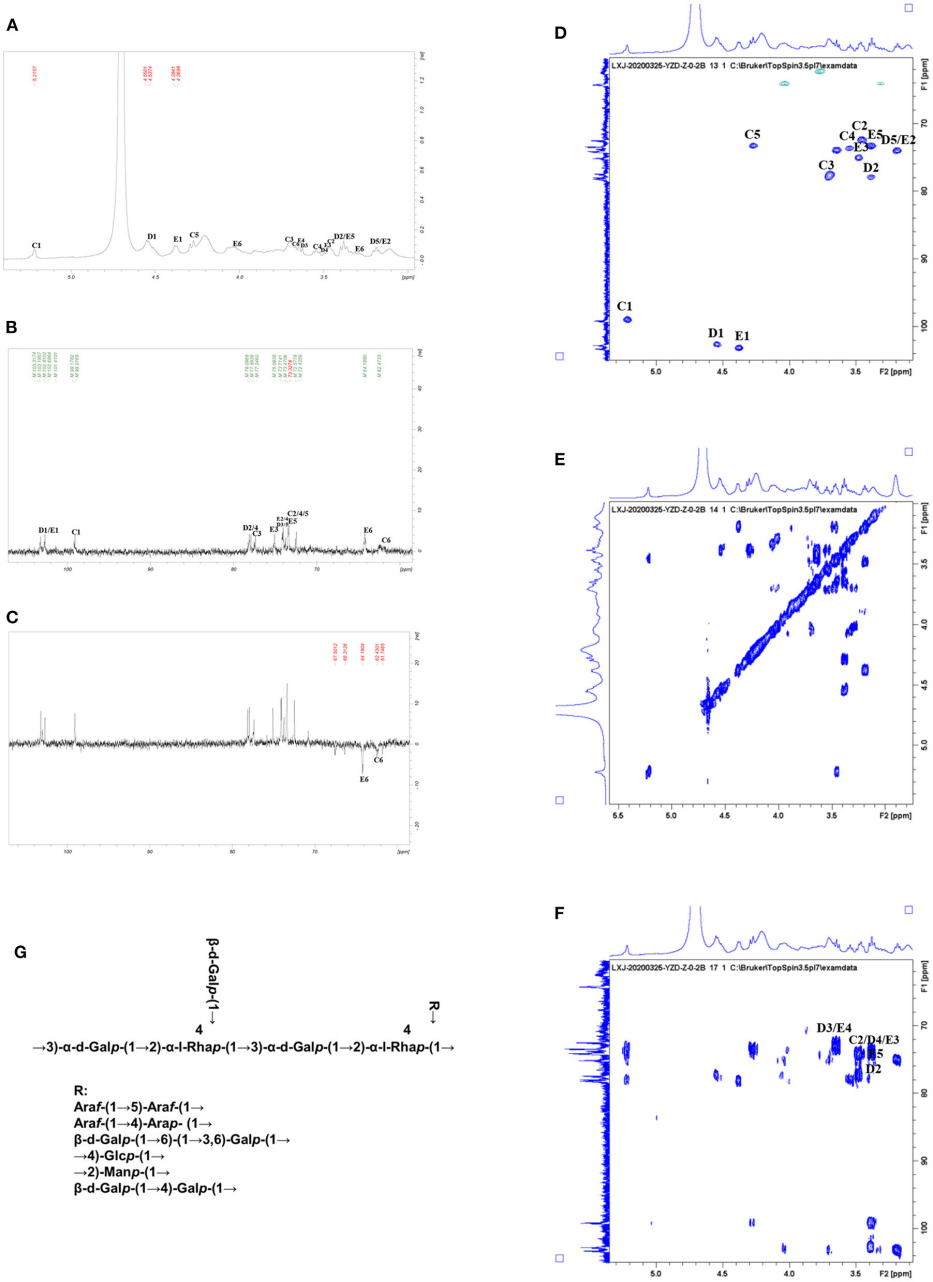
**(A)** ^1^HNMR, **(B)** ^13^C NMR, **(C)** DEPT-135, **(D)** HSQC, **(E)** HHCOSY, **(F)** HMBC spectra, and the structural formula **(G)** of CWP-0.2.

All NMR spectra of CWP and CWP-0.2 are shown in Figures 1, 2, respectively. The major chemical shifts are listed in Table 2. Signals were assigned using literature values (26, 27). 1D NMR spectra, including ^1^H, ^13^C and DEPT135 (Figures 2A–C, 3A–C), in CWP and CWP-0.2 had obvious differences. For the proton spectrum signals, the ^1^H-NMR signals of CWP mainly focused on 3.0–5.5 ppm (Figure 2A), while CWP-0.2 ^1^H-NMR signals were focused on 1–8 ppm (Figure 3A). The 0–3.2 ppm peaks were attributed to the hydrogen signals of aliphatic alkanes. The 3.2–5.5 ppm peaks belonged to the hydrogen signals of polysaccharides, while the 6.5–8 ppm peaks belonged to aromatic hydrogen signals. According to Figure 3A, CWP-0.2 was mainly composed of galactose, which was consistent with the details of Table 1. In the ^13^C NMR (126 MHz, D_2_O) carbon spectra, the ^13^C NMR signals of CWP and CWP-0.2 were both mainly concentrated from 60 to 120 ppm. As shown in Figure 2B of CWP, the main anomeric carbon signal peaks were δ105.54, 81.64, 77.81, 76.63, 64.74 and 61.44. For Figure 3B of CWP-0.2, the anomeric carbon signal peaks were mainly δ103.32, 103.20, 102.81, 102.69, 101.41, 99.18, 99.08, 78.10, 77.89, 77.35, 75.09, 73.71, 73.47, 73.33, 72.58, 72.44, 64.20 and 62.47. In the Dept135 spectra, for the CWP, we found that the methylene signal peaks δ 62.23 and δ 63.47 belonged to C1 and C6 of the fructose residues, respectively (Figure 2C). Regarding CWP-0.2, the peaks of 67.50, 66.31, 64.19, 62.43 and 61.75 were inverted, indicating that 67.50, 66.31, 64.19, 62.43 and 61.75 might be the chemical shifts of C6 (Figure 3C). Combined with the ^13^C NMR and Dept135 spectra of CWP-0.2 (Figures 3B,C), the methylene signal peaks were mainly δ 23.40-67.51 ppm, the peaks at 67.51, 66.32, 64.20, 62.44 and 61.76 ppm were the C6 signal peaks of sugar and 14.51, 19.93, 21.61 PPM were methyl signals. According to the HSQC (Figures 2D, 3D) and ^1^H-^1^HCOSY (Figures 2E, 3E) 2D spectra of CWP and CWP-0.2, the chemical displacements of ^1^H NMR and ^13^C NMR of the main residues were classified in Table 3. For the CWP, in HHCOSY analysis, three groups of chemical displacements located at 4.10/4.01, 4.01/3.87, and 3.87/3.48 represent the correlations of H3–H4, H4–H5, and H5–H6 on the fructose residue (Figure 2E). For the CWP-0.2, 99.12 ppm of anomeric carbon and 5.21 ppm of anomeric hydrogen were determined in the HSQC spectrum (Figure 3D). Then, according to HHCOSY (Figure 3E), the signals of H1–2, H2–3, H3–4, and H4–5 were 3.54/4.28, 5.21/3.46, 3.46/3.70, 3.70/3.54, and 3.54/4.28, respectively. Therefore, we inferred that H1, H2, H3, H4, and H5 of the signals were 5.21, 3.46, 3.7, 3.54 and 4.28 ppm, respectively. The corresponding C1–5 signals were 99.12, 72.5, 77.43, 73.43, and 73.17 ppm, and the chemical shift of C6 was 64.14. Therefore, the signal should go to → 3)-α-d-Gal*p*-(1 → . Similarly, connection modes of → 2,4)-α-l-Rha*p*-(1 → and β-d-Gal*p*-(1 → were also detected. HMBC 2D spectra analyses of CWP and CWP-0.2 were shown in Figures 2F, 3F. From the HMBC spectrum of CWP (Figure 2F), δ104.56 and δ3.60, 3.68 had correlation peaks, which were classified as C2 (β-d-Fru*f*-2,1)-H1a,b (β-d-Fru*f*-2,1), indicating → 2-β-d-Fru*f*-1 → 2-β-d-Fru*f*-1 →. At the same time, we also detected H3(β-d-Fru*f*-2,1)-C2 (β-d-Fru*f*-2,1), H5(β-d-Fru*f*-2,1)-C2 (β-d-Fru*f*-2,1). Meanwhile, the cross peaks of H4 (β-d-Fru*f*-2,1)-C5 (β-d-Fru*f*-2,1), H3 (β-d-Fru*f*-2,1)-C5 (β-d-Fru*f*-2,1) and H1a, b (β-d-Fru*f*-2,1)-C3 (β-d-Fru*f*-2,1) were observed in the HSQC scheme (Figure 2D). These results were consistent with the NMR analysis results. Regarding the HMBC spectrum of CWP-0.2 (Figure 3F), the anomeric carbon of → 3)-α-d-Gal*p*-(1 → and H2 of the → 2,4)-α-l-Rha*p*-(1 → correlation peaks were detected. Meanwhile, the anomeric hydrogen of → 3)-α-d-Gal*p*-(1 → and C2 of the → 2,4)-α-l-Rha*p*-(1 → correlation peaks were also detected. These two findings suggest the existence of → 3)-α-d-Gal*p*-(1 → 2,4)-α-l-Rha*p*-(1 →. Similarly, the correlation peaks, the anomeric carbon of → 2,4)-α-l-Rha*p*-(1 → and H3 of → 3)-α-d-Gal*p*-(1 →, the anomeric hydrogen of → 2,4)-α-l-Rha*p*-(1 → and C3 of → 3)-α-d-Gal*p*-(1 →, are consistent, indicating the presence of → 2,4)-α-l-Rha*p*-(1 → 3)-α-d-Gal*p*-(1 →. At the same time, the anomeric carbon of β-d-Gal*p*-(1 → and H4 of → 2,4)-α-l-Rha*p*-(1 → had correlation peaks, indicating the existence of β-d-Gal*p*-(1 → 2,4)-α-l-Rha*p*-(1 →.

**Table 3:** The major ^13^C NMR Chemical shift (ppm) for CWP and CWP-0.2.

**Polysaccharide**	**Glycosyl residues**	**H1a**	**H1b**	**H2**	**H3**	**H4**	**H5**	**H6a**	**H6b**
		**C1**	**C1**	**C2**	**C3**	**C4**	**C5**	**C6a**	**C6b**
CWP	Residue B: β-d-Fru*f*-2,1	3.6	3.68		4.1	4.01	3.87	3.48	3.82
		61.31		104.54	77.77	76.56	81.86	64.62	
CWP-0.2	Residue C: → 3)-α-d-Gal*p*-(1 →	5.21		3.46	3.7	3.54	4.28	3.66	3.66
		99.12		72.5	77.43	73.43	73.17	62.05	62.05
	Residue D: → 2,4)-α-d-Rha*p*-(1 →	4.54		3.39	3.64	3.48	3.19	1.27	
		102.8		77.92	73.99	77.67	74.04	17.71	
	Residue E: β-d-Gal*p*-(1 →	4.38		3.19	3.47	3.65	3.39	3.3	4.04
		103.15		74.04	75.09	73.99	73.25	64.14	

Therefore, the main chain connection of CWP was → (2-β-d-Fru*f*-1) n → (Figure 2G). Regarding the main chain connection of CWP-0.2, the → 2,4)-α-l-Rha*p*-(1 → 3)-α-d-Gal*p*-(1 → was backbone, and the branched chain was linked to the backbone by → 2,4)-α-l-Rha*p*-(1 → o-4 (Figure 3G). According to the results of monosaccharide composition and NMR, it can be confirmed that the polysaccharide is inulin polysaccharide.

### Antioxidant activities

It has been reported that plant polysaccharides could protect the body from oxidative damage (28). The antioxidant activities of CWP and CWP-0.2 were measured using DPPH· and ABTS·^+^ assays, which have been widely used in evaluating the antioxidant activities of various natural extracts. As shown in Table 4, all polysaccharide fractions exhibited antioxidant activity, and CWP-0.2 showed a higher scavenging activity against DPPH· and ABTS·^+^ than CWP. Yao et al. (9) reported that the scavenging activity of polysaccharides may be related to the molecular weight. Due to the lower molecular weight of CWP-0.2, it had a higher free radical scavenging capacity, both for DPPH· and ABTS·^+^ assays. Ye et al. (4) reported that polysaccharides from chickpea hulls (CHPS-1, CHPS-2, and CHPS-3) showed significant antioxidant activity against DPPH and ABTS free radicals, superoxide anion radicals and reducing power (4). All results suggested that polysaccharides from chickpea could be developed as potential antioxidants in the food, pharmacy, and cosmetic industries.

**Table 4:** The antioxidant activity of CWP and CWP-0.2.

**Polysaccharide**	**DPPH**·**free radical scavenging activity (TAEC**, μ**M/g)**	**ABTS**·^+^ **radical scavenging activity (TAEC**, μ**M/g)**
CWP	2.35 ± 0.39^b^	6.99 ± 0.44^b^
CWP-0.2	4.92 ± 0.95^a^	17.04 ± 0.87^a^

Data are expressed as mean ± standard. Means within each column with different lowercase letters are significantly different (p < 0.05)

### Immunoregulatory activities

**Figure 4:**
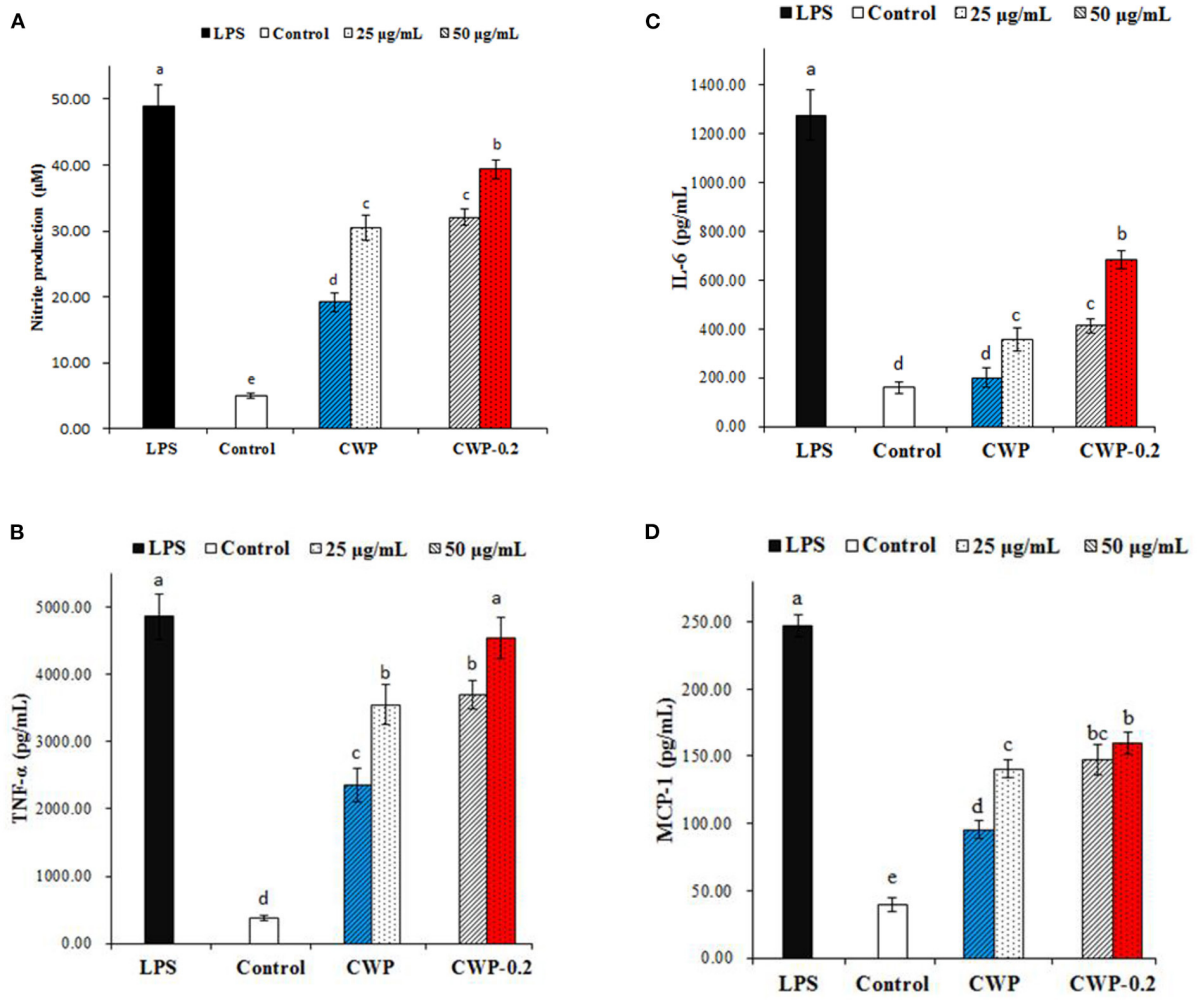
Effects of polysaccharides CWP and CWP-0.2 on RAW 264.7 macrophage **(A)** NO, **(B)** TNF-α, **(C)** IL-6, and **(D)** MCP-1 production. Values are the mean ± SD (*n* = 3). The different small letters in different columns represent significant difference at 0.05 level.

Nitric oxide (NO) is a significant signaling molecule in many tissues that play important roles in regulating varieties of physiological processes. Nitrite concentrations in the supernatant of polysaccharide-stimulated macrophages were determined as a reflection of NO production. Figure 4A showed the effects of different concentrations (25 and 50 μg/mL) of CWP and CWP-0.2 on the production of NO on RAW 264.7 cells. Compared with the control group, the production of NO significantly increased in CWP and CWP-0.2 in a dose-dependent manner at concentrations ranging from 25 to 50 μg/mL. In addition, CWP-0.2 showed significantly higher NO production than CWP at both concentrations, indicating a higher influence on macrophage activation.

Previous research has shown that activated macrophages play a significant role in mediating innate and adaptive immune responses via the production of a signaling chemical (NO) and cytokines (29). In this work, we also evaluated the effect of polysaccharides on the production of TNF-α, IL-6, and MCP-1 by RAW 264.7 macrophages, which were multifunctional cytokines associated with the production of NO and had a net effect on balancing its proinflammatory and immunosuppressive activities (30). As shown in Figures 4B–D, CWP or CWP-0.2 (25 and 50 μg/mL) treatment significantly increased the production of TNF-α, IL-6, and MCP-1 in RAW 264.7 cells in a concentration-dependent manner. CWP-0.2 showed stronger auxo-actions on TNF-α, IL-6, and MCP-1 production than CWP at both concentrations, which was similar to the production of NO. This might be because CWP-0.2, with its lower molecular weight, easily entered macrophages and even increased the strength of interactions between functional groups (hydroxyl or carboxylic groups) and proteins of RAW264.7 cells (29). Similar to these results, in our previous study, we obtained four fractions of water-extractable polysaccharides from adzuki bean, AAP-1 (94.2 kDa), AAP-1' (63.1 kDa), AAP-2 (82.3 kDa), and AAP-2' (60.4 kDa), and AAP-2', with the smallest molecular weight, showed the most potential activities and induced statistically higher NO production (29). It has been extensively shown that the immunomodulatory activity of polysaccharides was dependent on their chemical composition, molecular weight, conformation, glycosidic linkage, and degree of branching (31). However, studies on the immunoregulatory activities of chickpea polysaccharides were still limited. Further study should be focused on the immunoregulatory activities and the relationship between the immunoregulatory mechanism and the structure of chickpea polysaccharides.

## Conclusion

In conclusion, two polysaccharide fractions, were obtained and purified from chickpea seeds. Their molecular weight was determined to be 7.37 × 10^5^ Da and 1.58 × 10^4^ Da, respectively. Further structural characterization indicated that the main chain connection of CWP was → (2-β-d-Fru*f*-1) n →, and the main chain connection of CWP-0.2 was explored as → 2,4)-α-l-Rha*p*-(1 → 3)-α-d-Gal*p*-(1 → with the branched chain of → 2,4)-α-l-Rha*p*-(1 → o-4. The antioxidant and immunoregulatory activities of the two fractions were also demonstrated, and CWP-0.2 revealed significantly higher activity than CWP. These results will expand the knowledge of chickpea polysaccharides, and contribute to clarifying the benefits of chickpea foods.

## Data availability statement

The original contributions presented in the study are included in the article/supplementary material, further inquiries can be directed to the corresponding author/s.

## Author contributions

YZ, ZS, and YW contributed to conception and design of the study. YZ, ZS, YY, and BD organized the database. ZS and YW performed the statistical analysis. YZ wrote the first draft of the manuscript. ZS, YW, and BD wrote sections of the manuscript. All authors contributed to manuscript revision, read, and approved the submitted version.

## Funding

This work was supported by the Special National Key Research and Development Plan (2021YFD1600100), Key Laboratory of Grain Crop Genetic Resources Evaluation and Utilization, Chia Agriculture Research System of MOF and MARA (CARS-08-G21) (Food Legumes).

## Conflict of interest

The authors declare that the research was conducted in the absence of any commercial or financial relationships that could be construed as a potential conflict of interest.

## Publisher's note

All claims expressed in this article are solely those of the authors and do not necessarily represent those of their affiliated organizations, or those of the publisher, the editors and the reviewers. Any product that may be evaluated in this article, or claim that may be made by its manufacturer, is not guaranteed or endorsed by the publisher.
